# NF-κB Repression by PIAS3 Mediated RelA SUMOylation

**DOI:** 10.1371/journal.pone.0037636

**Published:** 2012-05-23

**Authors:** Yuangang Liu, Rebecca Bridges, Aaron Wortham, Molly Kulesz-Martin

**Affiliations:** Department of Dermatology, Oregon Health and Science University, Portland, Oregon, United States of America; Johns Hopkins School of Medicine, United States of America

## Abstract

Negative regulation of the NF-κB transcription factor is essential for tissue homeostasis in response to stress and inflammation. NF-κB activity is regulated by a variety of biochemical mechanisms including phosphorylation, acetylation, and ubiquitination. In this study, we provide the first experimental evidence that NF-κB is regulated by SUMOylation, where the RelA subunit of NF-κB is SUMOylated by PIAS3, a member of the PIAS (protein inhibitor of activated STAT) protein family with E3 SUMO ligase activity. PIAS3-mediated NF-κB repression was compromised by either RelA mutant resistant to SUMOylation or PIAS3 mutant defective in SUMOylation. PIAS3-mediated SUMOylation of endogenous RelA was induced by NF-κB activation thus forming a negative regulatory loop. The SUMOylation of endogenous RelA was enhanced in IκBα null as compared with wild type fibroblasts. The RelA SUMOylation was induced by TNFα but not leptomycin B mediated RelA nuclear translocation. Furthermore, RelA mutants defective in DNA binding were not SUMOylated by PIAS3, suggesting that RelA DNA binding is a signal for PIAS3-mediated SUMOylation. These results support a novel negative feedback mechanism for NF-κB regulation by PIAS3-mediated RelA SUMOylation.

## Introduction

NF-κB is a transcription factor that mediates cellular response to inflammation, immune response, and stress [1]. Deregulation of NF-κB is one of the common features in many pathological disorders including inflammatory diseases and cancer. NF-κB is a dimeric protein which can be comprised of a variety of combinations of Rel family DNA binding proteins including RelA (p65), RelB, c-Rel, p50, and p52. A heterodimer of RelA and p50 is the most common combination in the canonical NF-κB signaling pathway. In unstimulated cells, NF-κB is held in check by the inhibitor of NF-κB (IκBα) which sequesters NF-κB in the cytoplasm and prevents NF-κB DNA binding. Upon stimulation, IκBα is phosphorylated by IκB kinases, leading to its degradation. The degradation of IκBα allows the free NF-κB to translocate to the nucleus where it functions as a transcription factor to induce the expression of proinflammatory cytokines, chemokines, and factors for cell proliferation and survival [2].

Aberrant activation of NF-κB is detrimental to the host and may lead to a variety of inflammation related diseases like cancer, psoriasis and arthritis. Thus, as in many other signal transduction pathways, there are multiple feedback mechanisms to balance the activity of NF-κB. A well established mechanism is NF-κB dependent induction of IκBα which disrupts NF-κB DNA binding and shuttles nuclear NF-κB back to cytoplasm, thus forming a negative regulation loop [3], [4], [5]. A20 is another NF-κB induced gene that inhibits NF-κB activity by degrading receptor interacting protein (RIP), an essential mediator for the activation of the TNF receptor-associated signaling complex in the cytoplasm [6]. In addition to negative regulation by NF-κB inducible genes, NF-κB is negatively regulated by CYLD, a deubiquitinase that represses the activation of the IKK complex by removing K63-linked ubiquitin chains from TRAFs and NEMO [7], [8], [9].

In addition to protein ubiquitination, growing evidence suggests that several proteins in the NF-κB pathway are regulated by SUMOylation [10]. SUMOylation is a posttranslational modification involving covalent conjugation of small ubiquitin-like modifier proteins (SUMO) to target proteins. In contrast to protein ubiquitination, which generally tags proteins for proteasome-mediated degradation, SUMOylation modulates protein localization, protein/protein interaction, transcriptional regulation, as well as protein stabilization. SUMOylation of IκBα inhibits NF-κB activation by blocking IκBα ubiquitination and degradation [11]. In response to genotoxic stress but not inflammatory challenge, NF-κB is activated by PIASy-mediated NEMO SUMOylation [12].

Mammalian PIAS has four family members including PIAS1, PIAS2 (PIASx), PIAS3, and PIASy [13]. PIAS proteins have four conserved structural domains and motifs: a SAP domain for chromatin binding, PINIT motif for localization, SP-RING domain for E3-SUMO ligation, and a SUMO-interacting motif for SUMO binding. While PIASy-mediated NEMO SUMOylation contributes to NF-κB activation [12], PIAS1 and PIAS3 inhibit NF-κB activity by direct binding to the RelA subunit of NF-κB [14], [15]. PIAS1 binds to the C-terminal transactivation domain of RelA and blocks RelA binding to DNA *in vitro* and *in vivo* [14]. PIAS3 binds to the N-terminal DNA binding domain of RelA and interferes with RelA binding to the CBP coactivator [15]. In our previous studies, we also found that PIASy represses NF-κB activity in mouse keratinocytes and represses the expression of CCL20 chemokine in response to TNFα and/or IL-17A [16]. Although SUMO modification has been suggested as a mechanism for transcriptional repression conserved from yeast to human [17], RelA SUMOylation and its role in transcriptional regulation have not been defined.

In this study, we provide *in vitro* (cell free) and *in vivo* evidence that RelA is SUMOylated. RelA is predominantly SUMOylated by PIAS3, among PIAS family proteins. PIAS3-mediated NF-κB repression is compromised by either RelA mutant resistant to SUMOylation or PIAS3 mutant defective in SUMOylation. The SUMOylation of endogenous RelA by PIAS3 is induced by NF-κB activation. Furthermore, PIAS3-mediated RelA SUMOylation was dependent on RelA DNA binding. These data suggest PIAS3-mediated RelA SUMOylation as a novel negative regulatory mechanism for NF-κB regulation.

## Materials and Methods

### Plasmids

Flag-tagged RelA/p65 was constructed by cloning the RelA/p65 coding sequence from HA-tagged RelA (kindly provided by S. Ghosh, Yale University) into pCMVTag2B vector (Stratagene). V5-tagged RelA was constructed by cloning RelA coding sequence from Flag-tagged RelA vector into pCDNA-v5 vector (Invitrogen). RelA mutants (121/122K>R, 37K>R, 39E>I, 36Y>A) and catalytically dead PIAS3 mutant (299C>S) were generated by targeted mutagenesis (Stratagene). His-tagged RelA and PIAS3 were cloned into pET-24a expression vector for the production of PIAS3 and RelA recombinant protein in BL-21 *E. coli* cells. Flag-tagged Pias1and PIAS3 cDNA were kindly provided by Shaiu Ke, UCLA. His-tagged SUMO3 and constitutively active IKK2 were kindly provided by Paul Fraser [18] and Anjana Rao [19], respectively.

### Cell Culture and Transfection

Human embryonic kidney 293T (HEK293T) cells (Invitrogen), mouse 3T3 fibroblasts [20], IκBα null fibroblasts and wild type fibroblasts [3], [4], [5] were cultured in Dulbecco’s modified Eagle’s medium supplemented with 10% fetal calf serum. Cells were transfected in serum free OptiMEM medium (Invitrogen) at 90% confluence using Transfectin (Bio-Rad) according to the manufacturer’s instructions.

### Expression and Purification of Recombinant Bacterial Proteins

BL-21 cells containing the 6×his- and 6×his-Flag expression vectors noted above were grown to log phase in 300 ml Luria broth and induced with 1 mM IPTG (Fisher) for 1 hour. The cells were centrifuged at 5000 rpm for 5 mins at 4°C. The pellet was diluted in nickel lysis buffer (50 mM NaH_2_PO_4_, 300 mM NaCl, 20 mM imidazole, pH 8.0), treated with lysozyme (1 mg/ml) for 20 minutes on ice, and sonicated. The crude lysate was clarified by centrifugation at 10,000 rpm for 20 min. The lyaste was incubated with 1 mL of Ni-NTA agarose slurry (Invitrogen) in nickel lysis buffer for 1 hr at 4°C. The beads were washed three times with 6 ml nickel wash buffer (50 mM NaH_2_PO_4_, 300 mM NaCl, 60 mM imidazole, pH 8.0), and eluted in 500 µL fractions using the nickel elution buffer (50 mM NaH_2_PO_4_, 300 mM NaCl, 250 mM imidazole, pH 8.0). The eluants were dialyzed against DNAB buffer (50 mM NaCl, 20 mM Tris [pH7.2]; 4% glycerol; 0.1% Triton-100; 1 mM EDTA). The purity and concentration of purified proteins were verified by Coomassie stain and immunoblotting.

**Figure 1:**
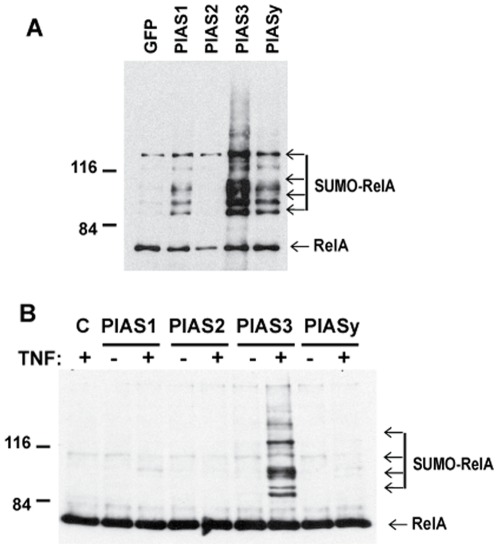
RelA is predominantly SUMOylated by PIAS3. A) SUMOylation of overexpressed RelA by PIAS proteins. Mouse 3T3 fibroblast cells were transfected with V5-tagged RelA, His-tagged SUMO3, and Flag-tagged PIAS vectors as indicated. SUMOylated RelA was measured by *in vivo* SUMOylation assay with anti-V5 antibody. B) TNFα-dependent SUMOylation of endogenous RelA in mouse 3T3 fibroblast cells. Mouse 3T3 cells were transfected with His-tagged SUMO3 and Flag-tagged PIAS vectors as indicated. The cells were treated with TNFα (20 ng/ml) for 4 hours after overnight transfection. SUMOylated RelA was measured by *in vivo* SUMOylation assay with anti-RelA antibody.

**Figure 2:**
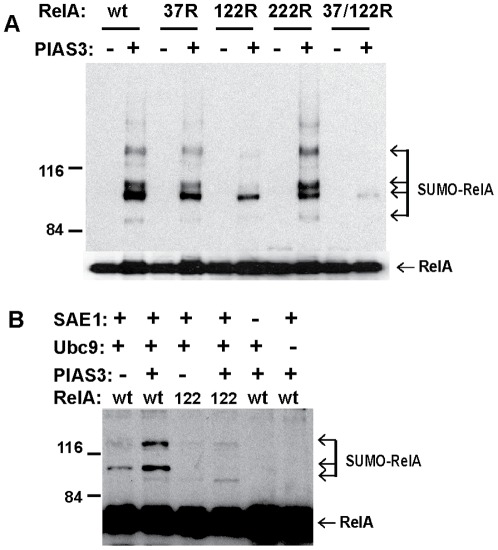
Identification of preferred RelA SUMOylation site. A) Mouse 3T3 cells were transfected with His-tagged SUMO3, v5-tagged RelA mutants 37R, 122R (121K>R/122K>R), and 222R as indicated, and Flag-tagged PIAS3 plasmids. The SUMOylated RelA was measured by nickel pull down followed by immunoblotting with V5 antibody. The total RelA protein was measured by direct immunoblotting with V5 antibody. B) Analysis of PIAS3-dependent RelA SUMOylation by *in vitro* SUMOylation assay. The *in vitro* SUMOylation assay was conducted with Purified E1 (SAE1), Ubc9, PIAS3, wild type RelA (wt), and RelA 122R mutant (122) as indicated. The SUMOylated RelA was detected by immunoblotting with anti-RelA antibody.

### *In vivo* SUMOylation Assay

Murine 3T3 cells were transfected with His6-tagged SUMO vector, V5-tagged RelA vector, and Flag-tagged PIAS vectors, and other vectors as indicated. 24 hrs after transfection, cells were washed twice with PBS and harvested in 1 ml of PBS. Fifty percent of cells collected were lysed in RIPA buffer and used for direct immunoblotting. The remainder was lysed in denaturing lysis buffer (6 ml of 6 M guanidinium-HCl, 0.1 M Na2HPO4/NaH2PO4, 0.01 M Tris/HCl, pH 8.0, 5 mM, imidazole and 10 mM beta-mercaptoethanol). 50 µl of Ni^2+^-NTA-agarose beads (Qiagen) were then added and lysates were rotated at room temperature (RT) for 4 h. The beads were washed for 5 min between each step at room temperature with each of the following buffers, successively: Buffer A (6 M guanidinium-HCl, 0.1 M Na2HPO4/NaH2PO4, 0.01 M Tris/HCl, pH 8.0 plus 10 mM b-mercaptoethanol); Buffer B (8 M urea, 0.1 M Na2HPO4/NaH2PO4, 0.01 M Tris/HCl, pH 8.0, 10 mM b-mercaptoethanol); and Buffer C (8 M urea, 0.1 M Na2HPO4/NaH2PO4, 0.01 M Tris/HCl, pH 6.3, 10 mM b-mercaptoethanol plus 0.2% Triton X-100). After the last wash His6-tagged SUMOylated products were eluted by incubating the beads in 75 ml of 200 mM imidazole, 0.15 M Tris/HCl pH 6.7, 30% glycerol, 0.72 M b-mercaptoethanol, 5%SDS for 20 min with vigorous shaking. The eluates were subjected to immunoblotting with anti-RelA antibody (C-20, Santa Cruz) or anti-V5 antibody (Abcam).

### *In vitro* SUMOylation Assay

His-tagged RelA, RelA 122R mutant, and PIAS3 proteins were produced in BL-21 *E. coli* transformed with the corresponding pET-24a expression vectors by IPTG induction for 1 hour at room temperature. The His-tagged proteins were purified by Ni-NTA affinity purification in lysis buffer (50 mM NaHPO4 pH8, 300 mM NaCl, 10 mM imidazole). The eluted proteins were dialyzed against binding buffer (50 mM Tris [pH7.2]; 4% glycerol; 0.1% Triton-100; 1 mM EDTA; 50 mM NaCl). The *in vitro* SUMOylation assay was performed in 40 µl of reaction buffer (20 mM HEPES [pH 7.4], 50 mM MgCl2, 20 mM ATP) with 0.1 µg of SAE1/SAE2 (Boston Biochem), 0.1 µg of Ubc9 (Boston Biochem), 6 ug of SUMO3, 15 ng of RelA, and 0.2 µg of PIAS3 at 37C for one hour. The reaction was terminated by 5× SDS sample buffer. The SUMOylated products were visualized by immunoblotting with anti-RelA antibody.

### Luciferase Assay

Mouse 3T3 cells were plated in 24 well plates and transfected with NF-κB reporter, and β-galactosidase reporter vectors. 24 hrs post-transfection, cells were lysed in luciferase assay buffer (0.1 M NaPO4 [pH 8.0], 4 mM ATP, 1 mM pyrophosphate, 1 mM MgCl, 20 mM DTT) supplemented with 0.2% Triton-100. Cleared supernatants were used for luciferase measurement. The β-galactosidase activity was measured by Tropix galacto-light beta-galactosidase assay (Applied Biosystems) for data normalization. The relative NF-kB luciferase activity was calculated by dividing the signal of NF-kB reporter by that of β-galactosidase.

### His-SUMO3 Lentivirus

His-SUMO3 [18] was cloned into XhoI/EcoRI sites of pSL35 lentiviral vector [21]. His-SUMO3 lentiviruses were generated using the four-plasmid system by cotransfection of 293T cells with pSL3, which expresses the vesicular stomatitis virus G envelope protein; pSL4, which expresses the HIV-1 *gag/pol* genes; pSL5, which expresses the *rev* gene; and pSL35 containing His-SUMO3. Lentivruses were harvested at 48 and 72 h after the transfection and concentrated by ultracentrifugation at 500,000×*g* for 90 min. The recombinant virus titer was determined for use of minimal viral particles to achieve ≥90% infection of IκBα null fibroblasts and wild type fibroblasts.

### DNA Affinity Immunoblot

RelA DNA binding activity was measured by DNA affinity immunoblotting, a sensitive *in vitro* technique for measurement of endogenous DNA binding proteins [22]. A 200 µg aliquot of nuclear extract was mixed with biotinylated NF-kappaB consensus binding sequence, biotin-CATAAGTCATGAGTTGAGGGGACTTTCCCAGGC in 1× DNA binding buffer [20 mM Tris (pH 7.2), 1 mM EDTA, and 0.06% Triton X-100 supplemented with 5 mM DTT, and 10 µg salmon sperm DNA), with a final NaCl level equal to 250 mM and glycerol level equal to 4%] in cold room for 30 minutes. The DNA bound proteins were captured by streptavidin magnasphere paramagnetic particles (Promega) and analyzed by immunoblotting.

## Results

### RelA SUMOylation by PIAS3

To determine whether RelA is SUMOylated by PIAS proteins, we evaluated *in vivo* RelA SUMOylation in murine 3T3 cells transiently transfected with V5-tagged RelA, Flag-tagged PIAS proteins, and His-tagged SUMO3. Analysis of nickel-affinity purified RelA from the cell lysate revealed several slowly migrating forms of RelA (Figure 1A). As the slowly migrating forms of RelA were dependent on co-transfection of his-tagged SUMO3 (Figure S1), they represent SUMOylated RelA species with one or more SUMO3 molecules. Although RelA SUMOylation by PIAS1 and PIASy was detectable, PIAS3 showed the most potent effect on RelA SUMOylation. PIAS3-dependent RelA SUMOylation was also detected in other cell types such as HEK293 and H1299 cells (Figure S2). To test whether endogenous RelA is SUMOylated by PIAS3, we evaluated RelA SUMOylation by *in vivo* SUMOylation assay in 3T3 cells transiently transfected with Flag-tagged PIAS and His-tagged SUMO3. The SUMOylation of endogenous RelA by either PIAS1 or PIASy was barely detectable (Figure 1B). The endogenous RelA was SUMOylated by PIAS3 only after NF-κB activation by treatment of cells with TNFα (Figure 1B). Taken together, these data provide evidence that RelA is SUMOylated and that RelA SUMOylation is mediated by PIAS3.

**Figure 3:**
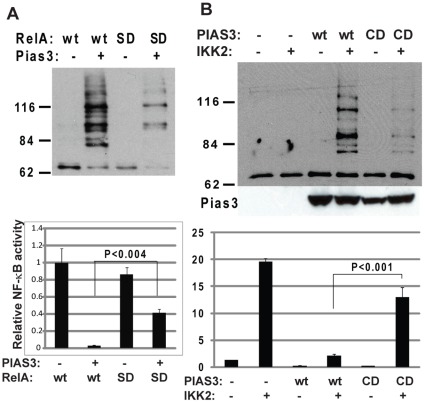
RelA SUMOylation-mediated NF-κB repression. A) Effects of RelA SUMO-dead mutation on PIAS3-mediated NF-κB repression. HEK 293T cells were transfected with his-tagged SUMO3, v5-tagged SUMO-dead mutant (SD, RelA with K>R mutations at 37, 121, and 122) and its wild type control (wt) in the presence or absence of Flag-tagged PIAS3. The SUMOylated RelA was measured by *in vivo* SUMOylation assay with anti-V5 antibody (upper panel). The corresponding NF-κB luciferase activity was measured (lower panel). B) Effects of PIAS3 catalytically dead mutation on PIAS3 mediated NF-κB repression. HEK 293T cells were transfected with wild type PIAS3 (WT), PIAS3 catalytically dead mutant (CD, PIAS3 with C>S mutation at 299), in the presence or absence of constitutively active IKK2. The SUMOylation of endogenous RelA was measured by *in vivo* SUMOylation assay with anti-RelA antibody (Upper panel). The corresponding NF-κB luciferase activity was measured (lower panel).

**Figure 4:**
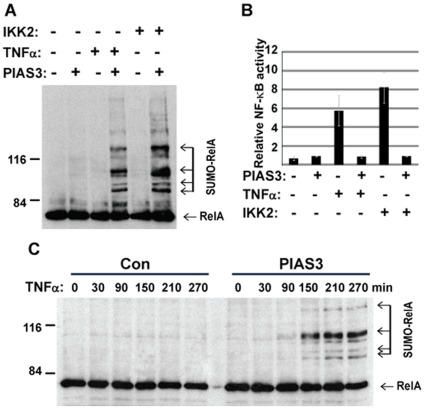
Endogenous RelA SUMOylation by PIAS3 is induced by NF-κB activation. A) HEK 293T were transfected with his-SUMO3 plus PIAS3 or GFP control plasmid as indicated. NF-κB activation was achieved by either co-transfection of constitutive IKK2 or TNFα treatment (20 ng/ml for 4 hours). SUMOylated RelA was measured by nickle pull down followed by immunoblotting with anti-RelA antibody. B) NF-κB repression by PIAS3. The NF-κB activity of cells described in panel A was measured by NF-κB luciferase assay. C) The time course of RelA SUMOylation in response to NF-κB activiation. HEK 293T cells were transfected with his-SUMO3 plus PIAS3 or control GFP plasmid as indicated. The cell lysates were collected for *in vivo* SUMOylation assay at indicated time points after TNFα treatment (20 ng/ml). SUMOylated RelA was detected by nickel pull down followed by immunoblotting with anti-RelA antibody.

**Figure 5:**
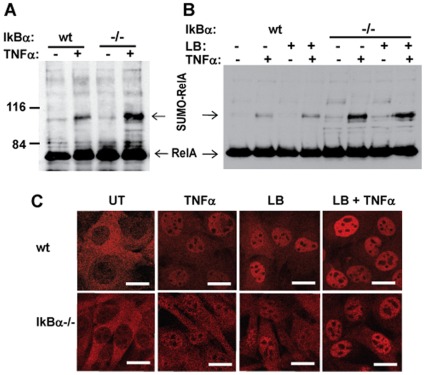
PIAS3-mediated RelA SUMOylation is enhanced by IκBα deficiency. IκBα null and wild type fibroblasts were stably transduced with His-tagged SUMO3 lentivirus. The SUMO3 transduced cells were treated with 20 ng/ml TNFα for 2 hours without (A) or with (B) 10 ug/ml leptomycin B for 2 hours, and subjected to *in vivo* SUMOylation assay. The SUMOylated RelA was detected by immunobloting with anti-RelA antibody. C) RelA localization in response to TNFα and/or leptomycin B treatment. RelA protein was stained with anti-RelA antibody. The bar equals 20 microns.

### Lysine 37, 121/122 are the Major SUMOylation Sites in RelA

Protein SUMOylation generally targets lysine residues in a ψK*X*E consensus sequence [23], where ψ is a hydrophobic amino acid residue, *X* represents any residue, E is an acidic residue, and K is a lysine residue to which SUMO moiety is covalently bound. There is no perfectly matched ψK*X*E consensus sequence in RelA protein. However, several nearly matched ψK*X*E consensus sequences in RelA protein are 37K(YKCE), 121/122K (VKKRD), and 222K (QKED). *In vivo* SUMOylation analysis of these RelA mutants revealed that PIAS3-mediated RelA SUMOylation was compromised by RelA mutations from lysine to arginine at 37 and 121/122 respectively (Figure 2A). PIAS3-mediated RelA SUMOylation was virtually abolished by compound mutation of 37K and 121/122K, indicating that lysine residues at 37 and 121/122 are involved in PIAS3-mediated RelA SUMOylation. To further define the role of PIAS3 in RelA SUMOylation, we conducted *in vitro* SUMOylation assays with purified RelA and PIAS3 proteins. As expected, RelA SUMOylation was abolished in the absence of either E1 enzyme (SAE1) or E2 enzyme (Ubc9) in the *in vitro* SUMOylation reaction. Consistent with the *in vivo* SUMOylation assays and RelA mutational analysis, RelA SUMOylation *in vitro* was enhanced by PIAS3 (Figure 2B) and RelA SUMOylation was compromised by 121/122 K>R mutation.

### RelA SUMOylation Dependent NF-kB Repression

Protein SUMOylation has been associated with a number of cellular activities including transcriptional repression by altering protein interactions [24]. To test the effects of PIAS3-mediated RelA SUMOylation, NF-κB luciferase activity was measured in 293T cells transfected with wild type RelA *and* SUMO defective RelA (SD-RelA, RelA with K>R mutations at 37, 121, and 122) (Figure 3A). NF-κB activation by wild type RelA was significantly repressed by PIAS3 overexpression. However, PIAS3-dependent NF-κB repression was compromised in the SUMOylation defective RelA mutant. As lysine is the accept site not only for SUMOylation but also for acetylation, methylation, and ubiquitination, these modifications may potentially contribute to altered NF-κB activity of SUMO defective RelA. To further define the contribution of RelA SUMOylation to NF-κB repression, NF-κB luciferase activity was compared in 293T cells transfected with wild type PIAS3 and catalytically dead PIAS3 mutant (CD-PIAS3, PIAS3 with C>S mutation at 299) (Figure 3B). The catalytically dead PIAS3 mutant failed to mediate RelA SUMOylation in response to NF-kB activation by constitutively active IKK2. These results suggest PIAS3-mediated RelA SUMOylation as a mechanism of NF-κB transcriptional repression.

**Figure 6:**
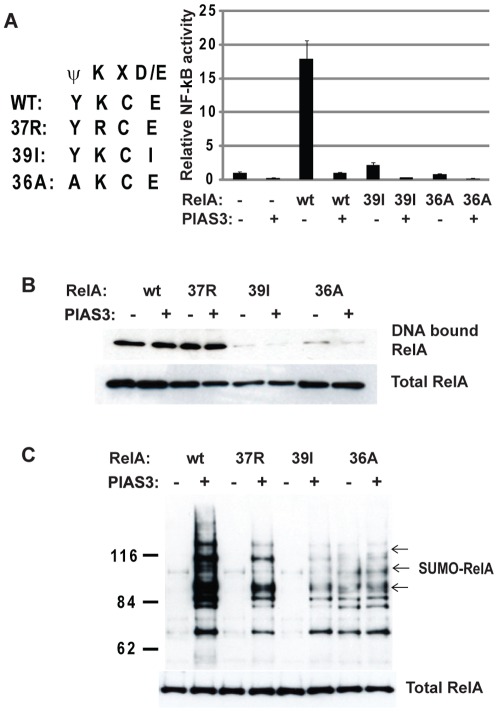
PIAS3-mediated RelA SUMOylation is dependent on RelA DNA binding. A) HEK 293T cells were transfected with Flag-tagged PIAS3, V5-tagged RelA and RelA mutants defective in DNA binding (39I and 36A) as indicated. The transactivation activity was measured by NF-κB luciferase activity. B) The nuclear extracts from HEK 293T cells transfected with indicated plasmids were subjected to DNA affinity immunoblotting with biotinylated NF-κB consensus binding DNA. DNA bound RelA was measured by immunoblotting with anti V5 antibody. C) The cell lysates from HEK293T cells transfected with indicated RelA mutants were collected for *in vivo* SUMOylation assay and detected by anti-V5 antibody for SUMOylated RelA (B).

### PIAS3 Mediated RelA SUMOylation is Induced by NF-κB Activation

Because PIAS3-mediated RelA SUMOylation was associated with NF-κB inhibition, we evaluated RelA SUMOylation as a potential mechanism for NF-κB negative regulation after activation by either TNFα treatment or co-transfection of constitutively active IKK2. Similar to TNFα treatment, the SUMOylation of endogenous RelA was induced by IKK2 co-transfection (Figure 4A). Analysis of NF-κB activity in the corresponding cells confirmed NF-κB activation by either TNFα treatment or IKK2 co-transfection (Figure 4B). In addition, the NF-κB activation by TNFα or IKK2 was repressed by PIAS3. This suggests that NF-κB activation is required for PIAS3-mediated RelA SUMOylation. Because NF-κB activation is repressed by PIAS3, RelA SUMOylation by PIAS3 is likely a mechanism for NF-κB negative regulation.

To further define the role of NF-κB activation in RelA SUMOylation, we examined the kinetics of PIAS3-mediated RelA SUMOylation in response to TNFα treatment. PIAS3-mediated RelA SUMOylation was observed 150 minutes after TNFα treatment and increased with time after TNFα treatment (Figure 4C). These results are consistent with RelA SUMOylation by PIAS3 as a negative regulatory mechanism for NF-κB.

### DNA Binding Dependent RelA SUMOylation by PIAS3

The central mechanism of NF-κB regulation is through the NF-κB negative regulator, IκBα, which sequesters NF-κB in the cytoplasm and dissociates NF-κB from DNA in the nucleus [2]. To further define the role of PIAS3-mediated RelA SUMOylation as a negative regulator of activated NF-κB in the nucleus, we evaluated RelA SUMOylation in cells with IκBα null background. To overcome low transfection frequency of IκBα null cells compared to wild type cells, we established cell lines with stable expression of His-tagged SUMO3. In the absence of TNFα, RelA SUMOylation was barely detected in either wild type or IκBα null cell lines. RelA SUMOylation was induced in both wild type and IκBα null cell lines upon TNFα treatment. IkBα null cells showed much stronger RelA SUMOylationon than its wild type counterpart (Figure 5A). To test whether enhanced RelA SUMOylation in IκBα null cells is associated with increased RelA nuclear accumulation or RelA DNA binding capability, we evaluated RelA SUMOylation in wild type and IκBα null cells treated with leptomycin B to block RelA nuclear export. Upon leptomycin B treatment, RelA accumulated in the nucleus in both wild type and IκBα null cells (Figure 5). However, RelA SUMOylation was detected only after TNFα treatment, not in either cell line treated with leptomycin B alone, showing that nuclear accumulation of RelA was insufficient for RelA SUMOylation.

Because dissociation of RelA DNA binding is another function of IκBα, we evaluated whether RelA DNA binding is required for PIAS3-mediated SUMOylation of RelA by using RelA mutants (39E>I and 36Y>A) defective in DNA binding [25]. The 39E>I and 36Y>A mutants, conferred defects in NF-κB activation as expected (Figure 6A). Consistent with previous DNA binding data [25], the39E>I and 36Y>A mutants failed to bind NF-κB consensus DNA binding sequence whereas the DNA binding activity of 37K>R mutant was not affected (Figure 6B). Compared with wild type RelA and SUMOylation compromised mutant at 37K, PIAS3-mediated RelA SUMOylation was abolished by both 39E>I and 36Y>A mutants (Figure 6B). It is of interest to note that weak RelA SUMOylation was induced independent of PIAS3 in the 36Y>A mutant (Figure 6C), likely due to the generation a perfectly matched SUMO consensus site (ψK*X*E), converting a polar amino acid to a hydrophobic amino acid. Even so, PIAS3-dependent RelA SUMOylation was abolished by the same mutation. The abolition of PIAS3-mediated RelA SUMOylation by different RelA mutants defective in DNA binding suggests that DNA bound RelA is the preferred target for SUMOylation by PIAS3.

## Discussion

NF-κB is known to be tightly negatively regulated by IκBα, which dissociates NF-κB from DNA and sequesters it in the cytoplasm [3], [4], [5], [26]. In this study, we provide experimental evidence for a novel mechanism for negative regulation of NF-κB by PIAS3-mediated RelA SUMOylation. PIAS3-mediated SUMOylation of endogenous RelA was observed to increase with time after TNFα stimulation, and RelA DNA binding-defective mutants were resistant to PIAS3-mediated SUMOylation. The dependence of SUMOylated RelA upon DNA binding capability as shown by DNA binding defective mutants, and upon TNFα stimulation and not simply nuclear localization after leptomycin B treatment, suggests a biochemical mechanism for NF-κB transcriptional repression.

PIAS3 has been reported to repress NF-κB activity through interfering with RelA binding to transcriptional co-activators [15]. However, PIAS3-mediated RelA SUMOylation was not considered a factor, largely due to the lack of perfectly matched SUMO consensus motifs in the RelA protein sequence and lack of detectable SUMOylated RelA under the conditions of that study. In our study, we demonstrate RelA SUMOylation by PIAS3 under both overexpressed and physiological conditions. While RelA could be weakly SUMOylated by other PIAS proteins like PIAS1 and PIASy, endogenous RelA was SUMOylated specifically by PIAS3, indicating PIAS3 as a primary mediator for RelA SUMOylation. Approaches to knockdown PIAS3 function by shRNA targeting of PIAS3 have been unsuccessful, so far, to address PIAS3dependence of RelA SUMOylation, in part possibly due to PIAS redundancy and shRNA efficiency. However, endogenous RelA SUMOylation specifically mediated by PIAS3 was demonstrated using the catalytically dead PIAS3 mutant.

Although protein SUMOylation is associated with many cellular activities, transcriptional repression is the primary consequence of protein SUMOylation. In fact, SUMO consensus sequence (ψK*X*E) was identified as a synergy control motif for transcriptional repression even before being recognized as a SUMO consensus sequence [27]. The identification of RelA SUMOylation has been hampered by the lack of perfectly matched ψK*X*E consensus sequence in RelA protein [15]. Recent global analysis of SUMOylated endogenous proteins revealed that one third of SUMO sites are not perfectly matched to ψK*X*E consensus sequences [28]. In this study, we have identified two imperfectly matched SUMOylation sites, 37K (YKCE), 121/122K (VKKRD) in RelA protein. Compound mutation of these sites abolished PIAS3-mediated RelA SUMOylation, and compromised PIAS3-mediated NF-κB repression (Figure 2 and Figure 3). Compromised NF-κB repression by PIAS3 mutant defective in E3 SUMO ligase activity further supported the role of PIAS3-mediated RelA SUMOylation in NF-κB repression (Figure 3B). These data suggest that RelA SUMOylation is a mechanism for NF-κB negative regulation, consistent with the known role of SUMOylation in transcriptional repression.

So far, the mechanism of RelA SUMOylation-mediated transcriptional repression is largely unknown. A number of transcriptional repressors and corepressors are either SUMOylated or associated with SUMOylated proteins through their SUMO interacting motif, thus enabling formation of transcription repression complexes [17], [26]. SUMOylation contributes to heterochromatin establishment and maintenance in yeast and Drosophila [29], [30]. A number of transcriptional repressors are either SUMOylated or capable of binding to the SUMO moiety through the SUMO interacting motif (SIM), including HDAC1 [31], CtBP [32], ZEB1 [26] and CoREST [33]. However, it has been a challenge to identify SUMOylation-dependent formation of transcription repression complexes. By sequential chromatin immunoprecipitation, Shuai’s group elegantly demonstrated PIAS1-dependent recruitment of heterochromatin protein 1 and DNA methyltransferase to the Foxp3 promoter [34]. These lines of evidence suggest that PIAS3-mediated RelA SUMOylation may provide a scaffold for the recruitment of transcriptional repressors with SUMO binding motifs thus leading to transcriptional repression. In addition to SUMOylation, the RelA SUMO sites are modified by methylation by SET7/9 [35] and acetylation by p300 and PCAF [36]. Further study to understand the relationship among these modifications is needed to define the mechanism of RelA SUMOylation in NF-κB transcriptional regulation.

Like many important signaling pathways, the NF-κB pathway is exquisitely regulated by multiple negative feedback regulatory mechanisms, such as the induction of IκBα to sequester NF-κB in the cytoplasm [5] and A20 to block NEMO activation [37]. In addition to NF-κB repression by RelA SUMOylation, we also demonstrated that PIAS3-mediated RelA SUMOylation was induced by NF-κB activation (Figure 5). The induction of RelA SUMOylation by NF-κB activation and the repression of NF-κB activity by RelA SUMOylation suggest that PIAS3-mediated RelA SUMOylation is a negative feedback mechanism for NF-kB regulation. This notion was further supported by the evidence from IκBα null cells, in which RelA SUMOylation was significantly enhanced, suggesting its compensatory role in NF-kB negative regulation.

It remains a challenge to define the molecular pathways that lead to RelA SUMOylation in response to NF-κB activation. The evidence from RelA mutants defective in DNA binding suggests that DNA-bound RelA is the preferred target for PIAS3-mediated RelA SUMOylation. In this study, RelA mutants (39E>I and 36Y>A) defective in DNA binding [25] also abolished PIAS3-mediated SUMOylation. Coincidently, both mutations reside in the N-terminal RelA SUMO consensus motif (36YKCE39). The abolition of PIAS3-mediated RelA SUMOylation by these mutations is very unlikely due to the disruption of the SUMO consensus site. Compared with the SUMOylation of 37K>R mutant, 39E>I mutation showed more severe effect on PIAS3-mediated RelA SUMOylation. Furthermore, 36Y>A mutation that generates a perfect SUMOylation motif, also abolished PIAS3-mediated RelA SUMOylation despite its enhancement of PIAS3-independent RelA SUMOylation. The abolition of PIAS3-mediated RelA SUMOylation by these various RelA DNA binding mutants suggests that RelA DNA binding is a determining factor in PIAS3-mediated RelA SUMOylation. *In vitro*, RelA DNA complex can be rapidly dissociated by IκBα, which reduces the half-life of the RelA DNA complex from 45 to 3 minutes [38]. RelA binding to DNA is negatively regulated by IκBα [3], which therefore reduces the pool of DNA-bound RelA available for SUMOylation. In the absence of IκBα negative regulation, increased DNA-bound RelA is available for SUMOylation. Thus, SUMOylation of RelA could be a mechanism to halt sustained NF-κB activation that is beyond the capacity of IκBα to control. The SUMOylation of DNA-bound RelA provides a molecular basis to form heterchromatic foci at promoters of genes regulated by NF-κB, to repress transcription, thus safeguarding against sustained transcriptional activation. Future studies will be needed to illustrate the mechanisms of RelA SUMOylation in NF-κB negative regulation in specific cell contexts, including defining NF-κB downstream genes affected by SUMOylation of RelA, and the associated transcriptional repressors in complex with SUMOylated RelA.

## Supporting Information

Figure S1 **PIAS3 mediated RelA SUMOylation is SUMO3 dependent.** 293T cells were transfected with v5 tagged RelA, flag-tagged PIAS, and his-tagged SUMO3 as indicated. SUMOylated RelA was measured by nickle pull down followed by immunoblotting with anti-V5 antibody.
(DOCX)

Figure S2 **RelA SUMOylation by PIAS3 in HEK293 and H1299 cells.** HEK293 cells (A) or H1299 cells (B) were transfected with V5-tagged RelA, His-tagged SUMO3, and Flag-tagged PIAS vectors as indicated. SUMOylated RelA was measured by nickle pull down followed by immunoblotting with anti-V5 antibody.
(DOCX)

## References

[1] Ghosh S, Karin M (2002). Missing pieces in the NF-kappaB puzzle..

[2] Hoffmann A, Baltimore D (2006). Circuitry of nuclear factor kappaB signaling.. Immunol Rev.

[3] Arenzana-Seisdedos F, Thompson J, Rodriguez MS, Bachelerie F, Thomas D (1995). Inducible nuclear expression of newly synthesized I kappa B alpha negatively regulates DNA-binding and transcriptional activities of NF-kappa B. Mol Cell Biol.

[4] Arenzana-Seisdedos F, Turpin P, Rodriguez M, Thomas D, Hay RT (1997). Nuclear localization of I kappa B alpha promotes active transport of NF-kappa B from the nucleus to the cytoplasm.. J Cell Sci 110 (Pt.

[5] Hoffmann A, Levchenko A, Scott ML, Baltimore D (2002). The IkappaB-NF-kappaB signaling module: temporal control and selective gene activation.. Science.

[6] Wertz IE, O’Rourke KM, Zhou H, Eby M, Aravind L (2004). De-ubiquitination and ubiquitin ligase domains of A20 downregulate NF-kappaB signalling.. Nature.

[7] Brummelkamp TR, Nijman SM, Dirac AM, Bernards R (2003). Loss of the cylindromatosis tumour suppressor inhibits apoptosis by activating NF-kappaB.. Nature.

[8] Kovalenko A, Chable-Bessia C, Cantarella G, Israel A, Wallach D (2003). The tumour suppressor CYLD negatively regulates NF-kappaB signalling by deubiquitination.. Nature.

[9] Trompouki E, Hatzivassiliou E, Tsichritzis T, Farmer H, Ashworth A (2003). CYLD is a deubiquitinating enzyme that negatively regulates NF-kappaB activation by TNFR family members.. Nature.

[10] Girdwood DW, Tatham MH, Hay RT (2004). SUMO and transcriptional regulation.. Semin Cell Dev Biol.

[11] Desterro JM, Rodriguez MS, Hay RT (1998). SUMO-1 modification of IkappaBalpha inhibits NF-kappaB activation.. Mol Cell.

[12] Mabb AM, Wuerzberger-Davis SM, Miyamoto S (2006). PIASy mediates NEMO sumoylation and NF-kappaB activation in response to genotoxic stress.. Nat Cell Biol.

[13] Shuai K, Liu B (2005). Regulation of gene-activation pathways by PIAS proteins in the immune system.. Nat Rev Immunol.

[14] Liu B, Yang R, Wong KA, Getman C, Stein N (2005). Negative regulation of NF-kappaB signaling by PIAS1.. Mol Cell Biol.

[15] Jang HD, Yoon K, Shin YJ, Kim J, Lee SY (2004). PIAS3 suppresses NF-kappaB-mediated transcription by interacting with the p65/RelA subunit.. J Biol Chem.

[16] Liu Y, Lagowski JP, Gao S, Raymond JH, White CR (2010). Regulation of the psoriatic chemokine CCL20 by E3 ligases Trim32 and Piasy in keratinocytes.. J Invest Dermatol.

[17] Garcia-Dominguez M, Reyes JC (2009). SUMO association with repressor complexes, emerging routes for transcriptional control.. Biochim Biophys Acta.

[18] Dorval V, Fraser PE (2006). Small ubiquitin-like modifier (SUMO) modification of natively unfolded proteins tau and alpha-synuclein.. J Biol Chem.

[19] Mercurio F, Zhu H, Murray BW, Shevchenko A, Bennett BL (1997). IKK-1 and IKK-2: cytokine-activated IkappaB kinases essential for NF-kappaB activation.. Science.

[20] Schneider BL, Bowden GT, Sutter C, Schweizer J, Han KA (1993). 7,12-Dimethylbenz[a]anthracene-induced mouse keratinocyte malignant transformation independent of Harvey ras activation.. JInvest Dermatol.

[21] Liu Y, Lagowski J, Sundholm A, Sundberg A, Kulesz-Martin M (2007). Microtubule disruption and tumor suppression by mitogen-activated protein kinase phosphatase 4.. Cancer Res.

[22] Liu Y, Asch H, Kulesz-Martin MF (2001). Functional quantification of DNA-binding proteins p53 and estrogen receptor in cells and tumor tissues by DNA affinity immunoblotting.. Cancer Res.

[23] Rodriguez MS, Dargemont C, Hay RT (2001). SUMO-1 conjugation in vivo requires both a consensus modification motif and nuclear targeting.. J Biol Chem.

[24] Hay RT (2005). SUMO: a history of modification.. Mol Cell.

[25] Wissink S, van Heerde EC, Schmitz ML, Kalkhoven E, van der Burg B (1997). Distinct domains of the RelA NF-kappaB subunit are required for negative cross-talk and direct interaction with the glucocorticoid receptor.. J Biol Chem.

[26] Wang J, Scully K, Zhu X, Cai L, Zhang J (2007). Opposing LSD1 complexes function in developmental gene activation and repression programmes.. Nature.

[27] Iniguez-Lluhi JA, Pearce D (2000). A common motif within the negative regulatory regions of multiple factors inhibits their transcriptional synergy.. Mol Cell Biol.

[28] Matic I, Schimmel J, Hendriks IA, van Santen MA, van de Rijke F (2010). Site-specific identification of SUMO-2 targets in cells reveals an inverted SUMOylation motif and a hydrophobic cluster SUMOylation motif.. Mol Cell.

[29] Shin JA, Choi ES, Kim HS, Ho JC, Watts FZ (2005). SUMO modification is involved in the maintenance of heterochromatin stability in fission yeast.. Mol Cell.

[30] Hari KL, Cook KR, Karpen GH (2001). The Drosophila Su(var)2–10 locus regulates chromosome structure and function and encodes a member of the PIAS protein family.. Genes Dev.

[31] David G, Neptune MA, DePinho RA (2002). SUMO-1 modification of histone deacetylase 1 (HDAC1) modulates its biological activities.. J Biol Chem.

[32] Lin X, Sun B, Liang M, Liang YY, Gast A (2003). Opposed regulation of corepressor CtBP by SUMOylation and PDZ binding.. Mol Cell.

[33] Ouyang J, Shi Y, Valin A, Xuan Y, Gill G (2009). Direct binding of CoREST1 to SUMO-2/3 contributes to gene-specific repression by the LSD1/CoREST1/HDAC complex.. Mol Cell.

[34] Liu B, Tahk S, Yee KM, Fan G, Shuai K (2010). The ligase PIAS1 restricts natural regulatory T cell differentiation by epigenetic repression.. Science.

[35] Li Y, Reddy MA, Miao F, Shanmugam N, Yee JK (2008). Role of the histone H3 lysine 4 methyltransferase, SET7/9, in the regulation of NF-kappaB-dependent inflammatory genes. Relevance to diabetes and inflammation.. J Biol Chem.

[36] Kiernan R, Bres V, Ng RW, Coudart MP, El Messaoudi S (2003). Post-activation turn-off of NF-kappa B-dependent transcription is regulated by acetylation of p65.. J Biol Chem.

[37] Werner SL, Kearns JD, Zadorozhnaya V, Lynch C, O’Dea E (2008). Encoding NF-kappaB temporal control in response to TNF: distinct roles for the negative regulators IkappaBalpha and A20.. Genes Dev.

[38] Zabel U, Baeuerle PA (1990). Purified human I kappa B can rapidly dissociate the complex of the NF-kappa B transcription factor with its cognate DNA.. Cell.

