# Supplementary figures and images for: NF-κB Repression by PIAS3 Mediated RelA SUMOylation
Source: PLoS One. 2012 May 23;7(5):e37636. doi: 10.1371/journal.pone.0037636 (PMC3359287; doi:10.1371/journal.pone.0037636)

**Supplementary information:**

Figure 1S

| 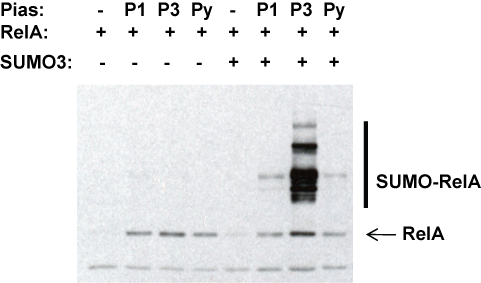 |
| --- |

Supplement: Figure S1 — PIAS3 mediated RelA SUMOylation is SUMO3 dependent. 293T cells were transfected with v5 tagged RelA, flag-tagged PIAS, and his-tagged SUMO3 as indicated. SUMOylated RelA was measured by nickle pull down followed by immunoblotting with anti-V5 antibody. (DOCX) [file pone.0037636.s001.docx]

**Supplementary information:**

Figure 2S

| 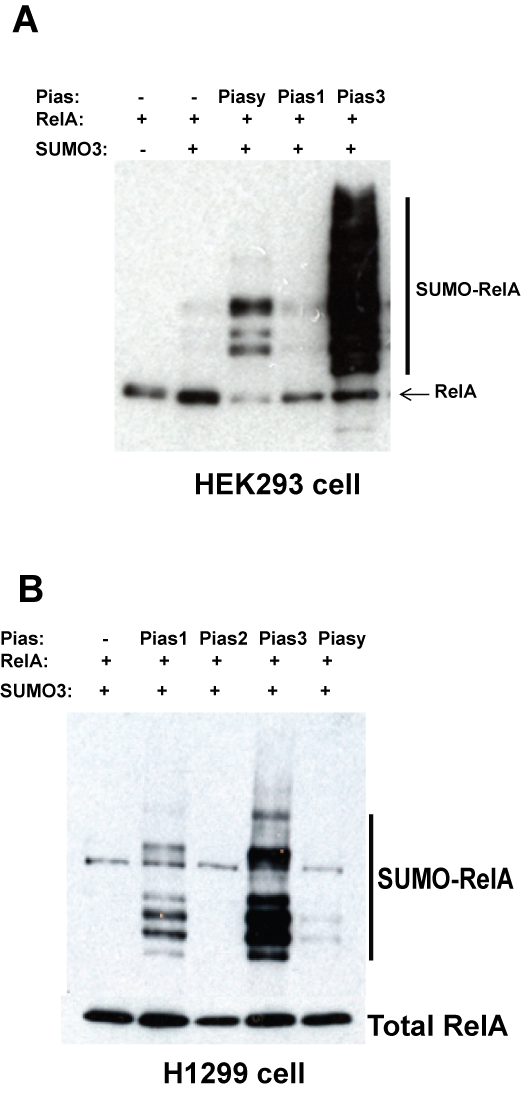 | 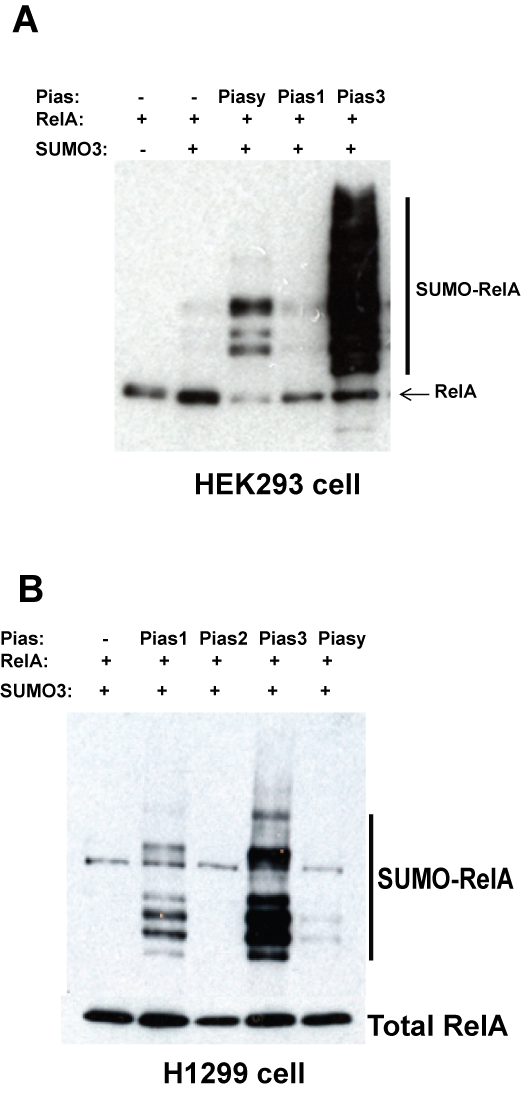 |
| --- | --- |
|  | |

Supplement: Figure S2 — RelA SUMOylation by PIAS3 in HEK293 and H1299 cells. HEK293 cells (A) or H1299 cells (B) were transfected with V5-tagged RelA, His-tagged SUMO3, and Flag-tagged PIAS vectors as indicated. SUMOylated RelA was measured by nickle pull down followed by immunoblotting with anti-V5 antibody. (DOCX) [file pone.0037636.s002.docx]
